# The government’s environmental attention and corporate green innovation: A threshold analysis and quantile regression approach

**DOI:** 10.1371/journal.pone.0311154

**Published:** 2024-10-31

**Authors:** Guoyan Huang, Xiao Li, Zhen Chu

**Affiliations:** School of Finance and Business, Shanghai Normal University, Shanghai, China; Huaqiao University, CHINA

## Abstract

Based on an analysis of 643 listed firms in clean technology sectors, this study explores the nonlinear impact of the government’s environmental attention (GEA) on firms’ green innovation by exploiting threshold and quantile regression techniques on Stata 17. We show that a double threshold exists when the level of the GEA is 51 or 104, above which the positive impact of the GEA on green innovation in cleantech firms significantly diminishes. The results from the quantile regression further indicate that cleantech firms receive almost no benefits from the GEA at lower levels of green innovation. Thus, policy-makers designing environmental policies should consider that the marginal benefit of environmental attention on green innovation wanes beyond certain levels, especially for firms that lack sufficient enthusiasm for innovation.

## Introduction

Climate change, air pollution, energy depletion, and other environmental issues have attracted substantial attention of governments worldwide. Many countries have introduced and enacted strategies to minimize the impact of environmental issues [1]. Environmental pollution, a severe human-induced environmental issue, has a negative externality that affects the overall welfare of society. Research shows that a variety of factors can mitigate environmental problems, such as digital technology and green innovation [2–4]. Green innovation, which can be defined as creating new products or technologies to reduce environmental risks [5], contributes to alleviating environmental pollution problems [6]. However, enterprises lack incentives for green innovation because its positive externality prevents them from fully benefiting from R&D results [7]. Externalities distort the relationship between costs and benefits for market players, leading to market inefficiencies or even failure, and government intervention has been considered a solution to this kind of problem. Externalities distort the relationship between costs and benefits for market participants, which can result in market inefficiency or failure. In response to this type of issue, government intervention has been proposed as a potential solution [8].

In recent years, the government’s environmental attention (GEA) has gradually become a new perspective in the field of government intervention [9]. “Attention” in “government’s attention” refers to a manager’s selective focus on specific information at the expense of others [10]. The government’s decision making depends on allocating its attention, a scarce resource in effect [11–13]. Governments are paying increasing attention to environmental issues [9] and address market failures in two ways: increasing the intensity of the environmental regulations and providing financial support to those who carry out green innovations. Strengthening environmental regulations increases firms’ costs by internalizing pollution costs, which many studies have argued inhibits firms’ green innovation [14, 15]. Cao et al. [16] discovered that the pressure of political promotion exerts a restraining influence on advancements in renewable energy technology, notably in hydropower tech innovation. In addition, some scholars have argued that environmental regulatory instruments, such as pollution charge, can improve green technological innovation in firms [17]. Government financial support, such as subsidies and tax incentives, can encourage R&D and promote green innovation among enterprises [18, 19]. These differences can be attributed to the potential nonlinearity in the connection between government intervention and corporate green innovation, as well as the heterogeneity in interfirm green innovation. For example, Zhang et al. [20] find that high financial subsidies are conducive to promoting real green technological innovation by environmental regulation, implying that the interaction between modes of government intervention makes the impact of government behavior on green innovation more complex. This paper empirically addresses these concerns in two specific ways. First, we employ a panel threshold model to compute nonlinear estimates for the sample of listed Chinese cleantech enterprises, which are primarily responsible for implementing green technologies. Second, we examine whether the derived estimates differ over the range of green innovation by employing an unconditional quantile regression model. Based on data from 634 listed cleantech companies in China, this paper explores the nonlinear impact of the government’s environmental attention (GEA) on firms’ green innovation by exploiting threshold and quantile regression techniques.

The following are the marginal contributions of this study. First, we attempt to incorporate the theory of the government’s attention into the research framework of corporate green innovation. This research logically articulates how the GEA influences green innovation in corporations and empirically demonstrates the nonlinear relationship that exists between the GEA and corporate green innovation through the threshold approach. Specifically, we find that the positive impact of the GEA on green innovation in cleantech firms significantly diminishes when the GEA is above the threshold values. Second, we provide insights into the variability of the impact of the GEA on cleantech firms with different levels of green innovation, which is rarely discussed in existing studies. We find that cleantech firms receive almost no benefit from the GEA at lower levels of green innovation. Third, we also probe the difference in response to GEA between substantive and strategic green innovation in cleantech enterprises, furnishing empirical evidence for studying the selection of types of innovation. This study’s main policy implication is to better understand the heterogeneous impact of the GEA on green innovation in firms. Policy-makers designing environmental policy should consider that the marginal benefit of the GEA for green innovation wanes beyond certain levels, particularly for firms that lack adequate enthusiasm for innovation.

The subsequent sections of this paper are structured as follows. The “Literature review” section reviews the literature on related topics and details the relationship between the GEA and corporate green innovation. The “Research design” section describes this article’s research design, including the data sources, variable measurements, and model settings. The “Empirical results” section describes the empirical analysis process and results. The “Robustness tests” section provides a series of robustness checks. The “Further analysis” section explores the effects of the GEA on substantive and strategic green innovation in cleantech firms. The “Conclusion” section provides the main conclusions and corresponding policy implications of this article.

## Literature review

The GEA has gradually emerged as an indicator of government intervention in the environment [9]. The “attention” in the “government’s attention” refers to the selective focus of governments on specific information at the expense of others [10]. Governments’ decision-making process depends on the allocation of their attention [11–13]. How governments express their concerns about public affairs varies widely from country to country: in the UK, the government has clarified to the public its administrative priorities and rationale through the “Speech from the Throne” [21, 22]; in the US, the annual State of the Union address reveals the international policy priorities of American policy-makers [23]. Similarly, in China, local governments express the content and focus of their work for the year to the public through government annual reports (GARs) [24]. A higher number of statements about the ecological environment in the GARs that are mentioned is associated with stronger government concern about this field. Generally, stronger government concern for the ecological environment is associated with greater intensity of environmental regulation and more financial support being given to the field of environmental protection, both of which affect firms’ green innovation.

Governments resort to environmental regulations to address the market failure caused by the negative externalities of environmental pollution, which internalizes the external costs of polluting firms. Porter and Linde [25] claimed that appropriate environmental regulations could increase enterprise productivity by leading to greater innovation, thus offsetting the costs of environmental regulations and increasing the profitability in the marketplace; this is the Porter hypothesis. Many studies have supported the Porter hypothesis and have provided empirical evidence that environmental policies can promote green innovation [26]. However, some scholars have held that government intervention in the environmental sector undermines corporate green innovation. They have argued that environmental regulations burden firms with incremental costs, thus not facilitating technological innovation [14, 27]. According to Chen [28], implementing a more stringent environmental standard may result in an increase in overall environmental quality. However, this approach can also limit a firm’s ability to provide diverse goods to various market segments [28]. Gouda et al. [29] discovered that even when composite legislation with strict standards is in place, the presence of positive synergies does not always ensure greater environmental quality unless significant scale efficiencies exist. Hafezi and Zolfagharinia [15] demonstrated that stringent rules might discourage enterprises from engaging in innovation, leading them to provide a uniform product instead of developing diverse offerings for various market niches. Governmental provision of increased financial assistance may have a significant influence on green innovation, as may the implementation of environmental rules. Green innovation has positive externalities [7], for which innovative enterprises cannot enjoy all of the dividends from technological innovation activities and find it challenging to maintain their competitive advantage in the industry. Government financial support, such as subsidies and tax incentives, can compensate for the losses incurred by enterprises through the positive externalities of innovation, thus promoting green innovation [18, 19]. However, according to the theory of organizational slack, additional government support is not needed. Resources beyond the minimum required for an organization to sustain operations are redundant [30] and detrimental to an organization’s innovation decisions [31]. The Porter hypothesis suggests that higher GEA may have a positive effect on green innovation in cleantech firms, whereas the theory of organizational slack suggests that this positive effect may decay. In conclusion, studies on the impact of government intervention on corporate green innovation have reported different findings, revealing that the relationship may be non-linear. It has been noted that government interventionist behavior in the environmental sector can have a non-linear facilitating effect on firms’ green innovation [32, 33]. However, few studies have focused on the non-linear effects of government intervention on firms’ green innovation from the perspective of the GEA. Therefore, we posit that financial support from increasing the GEA inspires corporate green innovation; however, the negative impacts of enhancing environmental regulations strengthen as the GEA continues to grow, thus diminishing its positive effect.

There may be considerable variations in the relationship between the GEA and corporate green innovation across firms. First, the beneficial effects of increasing the GEA are less noticeable for corporations with lower green innovation. Firms with low-level green innovation tend to be in the growth phase, and their ability to transform government-supported resources into a profit advantage is weaker [34], whereas high R&D intensity firms may benefit from economies of scale in patent development or from value-adding spillovers inside the corporation [35]. Through simulation, Xiao and Li [18] found that the evolution of green innovation paths for firms with innovation advantages is more affected by government green innovation subsidies. Second, the negative effects of increasing the GEA are more noticeable for corporations with a lower level of green innovation. Enhanced environmental regulations impose a cost burden on firms, which can be compensated for by the benefits from green innovation; therefore, firms with low innovation experience a minor compensatory effect [36]. Wu et al. [37] show that the effect of R&D subsidies on firms’ innovation is related to the firms’ R&D level, manifested in the fact that R&D subsidies inhibit the innovation of firms with low R&D levels and promote the innovation of firms with high R&D levels, which also suggests that there may be heterogeneity in the impact of government intervention behavior on firms’ innovation. Thus, we posit that the positive effect of growing the GEA on firms’ green innovation is weaker among firms with lower innovation.

A large number of studies have discussed the linear impact of government behavior on corporate green innovation from the perspectives of environmental regulation and financial support, providing empirical support for us to study the impact of the GEA on corporate green innovation. However, the controversy between the findings illustrates that the relationship between the GEA and corporate green innovation may be nonlinear and vary across firms with different levels of innovation. Insufficient empirical research has been conducted to examine the variability of this effect across varying increases in the GEA and corporate green innovation levels.

In China, cleantech enterprises play an essential role in promoting green innovation [38]. Cleantech enterprises are more motivated to develop green innovations to build their core competencies and are more vulnerable to government actions than other enterprises [39]. Based on these characteristics of cleantech firms, this paper takes cleantech firms as the object of study to better capture the typical and general relationship between the GEA and green innovation in corporations. We apply the threshold model proposed by Hansen [40] and provide nonlinear estimations of the GEA that elucidate the connection between the GEA and corporate green innovation. Subsequently, we refer to [41] and use the unconditional quantile regression (UQR) model to investigate the variations in these estimates over the range of corporation innovation levels.

## Research design

### Data and variables

We select of our sample using the following criteria.

First, we select listed cleantech companies in China as the sample companies; these companies are major contributors to green innovation in China and are highly dependent on policy support [38, 39]. Cleantech is a technology that avoids environmental damage at the source by using materials, processes, or practices to eliminate or reduce the creation of pollutants or wastes [42]. In 2021, the National Bureau of Statistics of China developed *The Statistical Classification of Energy Saving and Environmentally Clean Industries (2021)*, which offers the national economic industry codes available for reference for the cleantech industry. This document offers codes of three industries related to the cleantech industry, namely the energy-saving and environmental protection industries, clean production industries, and clean energy industries, as well as the industry codes for the corresponding sub-industries of these industries. We finalize the list of listed cleantech companies by matching the national economy industry codes.

Second, samples that were ST, *ST, or delisted during the sample period are omitted from our research. We utilize unbalanced panel data, including 6683 firm-year observations from 205 cities and 634 listed cleantech enterprises engaged in creative activities. The data span the period from 2001 to 2021 and are extracted from the WinGo Textual Analytics Database, the Chinese Research Data Services Platform (CNRDS), and the China Stock Market & Accounting Research (CSMAR) database.

The threshold variable and core explanatory variable are the government’s environmental attention (GEA). Textual analysis is an excellent way to overcome the problem of variable endogeneity [43] and is a common method for quantifying the government’s attention. The length of GARs has a negligible effect on the frequency of environment-related words as the contents of GARs are highly condensed. Therefore, we quantify the GEA using the frequency of environment-related keywords in GARs [44]. The complete indicator construction process is shown in S1 Appendix. The dependent variable, corporate green innovation (GA), is quantified by taking the logarithm of the number of green patent applications plus one, which is in reference to Li and Xiao [45]. We obtain the data of corporate green patent applications from the CNRDS database. This database verifies the “greenness” of a patent by comparing its International Patent Classification (IPC) with the IPC listed in the Green Catalogue of the World Intellectual Property Organization (WIPO) from 2010. Furthermore, a number of control variables are chosen to encompass business-specific attributes that influence corporate innovation, according to previous research [46]. Specifically, corporate-level characteristics are controlled by the length of the existence of the enterprise (Age); the logarithm of the enterprise’s total assets (Size); the ratio of current assets to current liabilities (CR); the ratio of total revenue to total assets (ROA); the ratio of operating costs to average inventory (ITR); and the product of return on equity and retained earnings r, which denotes the self-sustainable growth rate (SGR) of an enterprise. Table 1 presents the description and summary statistics of the variables.

**Table 1:** Variable description and summary statistics.

Variables	Description	Observations	Mean	S.D.	Min	Median	max
GEA	Government’s environmental attention	5976	61.48	19.81	1	61	149
GA	Logarithm of the number of green patent applications plus one	5976	1.140	1.330	0	0.693	6.931
GAst	Logarithm of the number of substantive green patent applications plus one	5976	0.785	1.150	0	0	6.752
GAsg	Logarithm of the number of strategic green patent applications plus one	5976	0.786	1.067	0	0	5.656
Age	Length of the existence of the enterprises (unit: year)	5976	14.97	5.935	0	15	41
Size	Logarithm of enterprises’ total assets	5976	22.01	1.429	19.34	21.73	28.27
CR	Ratio of current assets to current liabilities (unit: %)	5976	2.721	3.437	0.0747	1,753	78.51
ITR	Ratio of operating costs to average inventory (unit: %)	5976	111.9	3,229	0	3.584	199,494
ROA	Ratio of total revenue to total assets (unit: %)	5976	0.0394	0.081	-1.648	0.043	0.829
SGR	Product of return on equity and retained earnings ratio (unit: %)	5976	-0.0403	6.412	-494.7	0.051	5.718
CGDP	Logarithm of the city-level gross domestic product	5913	18.07	1.153	13.96	18.21	19.88
CTL	Logarithm of the expenditures in the science of cities	5922	12.68	1.983	5.318	12.88	15.53
CEL	Logarithm of the education expenditures of cities	5922	14.30	1.211	10.12	14.28	16.26

### Model construction

We use the fixed-effect threshold regression model to identify the possible existence of nonlinearity in the relationship between the GEA and green innovation in enterprises. Hansen [40] presented the fixed-effect panel threshold model, which specifies a single threshold model as follows:

$$\begin{array}{c} {GA}_{it}=\alpha_{0}+\alpha_{1}{GEA}_{kt}I({GEA}_{kt}\leq \gamma )+\beta_{2}{GEA}_{kt}I({GEA}_{kt}>\gamma )\\ +\sum_{j=1}^{n}\beta_{j}X_{it}+u_{i}+\varepsilon_{it} \end{array}$$ (1)

where *GEA*_*kt*_ denotes the GEA in city *k* at time *t* and *GA*_*it*_ indicates the degree of green innovation for firm *i* at time *t*. ***X***_*it*_ represents a collection of control variables at the company level. The parameters *u*_*i*_ and *ε*_*it*_ represent fixed effects particular to each firm and error term, respectively. The threshold parameter γ divides the cities into two regimes distinguished by the indicator function *I*(⋅). The null hypothesis of the model posits the absence of a threshold, whereas the alternative hypothesis asserts the presence of at least one threshold. This is summarized as follows:

$$H_{0}:\beta_{1}=\beta_{2}H_{a}:\beta_{1}\neq \beta_{2}$$

Rejecting the null hypothesis in the single threshold regression model results in the identification of multiple thresholds, which may be calculated for up to three thresholds. In the case of multiple thresholds, the models can be expanded from model (1) in the following manner:

$$\begin{array}{c} {GA}_{kt}=\alpha_{0}+\alpha_{1}{GEA}_{kt}I({GEA}_{kt}\leq \gamma_{1})+\alpha_{2}{GEA}_{kt}I(\gamma_{1}<{GEA}_{kt}\leq \gamma_{2})\\ +\alpha_{3}{GEA}_{kt}I({GEA}_{kt}>\gamma_{2})+\sum_{j=1}^{n}\beta_{j}X_{it}+u_{i}+\varepsilon_{it} \end{array}$$ (2)

$$\begin{array}{l} {GA}_{kt}=\alpha_{0}+\alpha_{1}{GEA}_{kt}I({GEA}_{kt}\leq \gamma_{1})+\alpha_{2}{GEA}_{kt}I(\gamma_{1}<{GEA}_{kt}\leq \gamma_{2})\\ \;+\alpha_{3}{GEA}_{kt}I(\gamma_{2}<{GEA}_{kt}\leq \gamma_{3})+\alpha_{4}{GEA}_{kt}I({GEA}_{kt}>\gamma_{3})\\ \;+\sum_{j=1}^{n}\beta_{j}X_{it}+u_{i}+\varepsilon_{it} \end{array}$$ (3)

Model (2) is used to estimate the threshold parameter γ and coefficient β for a double threshold regression, and Model (3) is used to estimate the same parameters for a triple threshold regression. Based on these parameters, the sample is separated into distinct regimes.

Additionally, we employ the quantile regression model (QRM) to examine the heterogeneous effects of GEA on corporations with varying degrees of green innovation. More precisely, the control factors cannot affect the unconditional quantile regression model (UQRM), ensuring a more consistent definition of quantiles. Therefore, the UQRM is more suitable for addressing many control variables [47]. We cite the study conducted by Firpo, Fortin [41] and implement the UQR model in the following manner:

$$RIF(Y_{it};q_{\tau },F_{Y})=\beta_{0}+\beta_{\tau 1}{GEA}_{kt}+\sum_{j=1}^{n}\beta_{\tau j}X_{it}+u_{i}+\varepsilon_{it}$$ (4)

where *β*_*τ*1_ represents the marginal impacts of GEA on companies in the green innovation quantile *q*_*τ*_; *u*_*i*_ denotes corporate-specific fixed effects; and *ε*_*it*_ denotes error terms; *τ*∈[0.1,0.9].

## Main results

### Threshold regression results

In this section, we ascertain the presence of single, double, and triple thresholds by estimating Models (1), (2) and (3), respectively. Tables 2 and 3 present the threshold regression results. The statistical significance of the numerous criteria was assessed, and the results are presented in Table 2. According to Table 2, the F statistic for the single threshold is 11.24, exceeding the crucial value of 8.771. This indicates that the single threshold is statistically significant at the 5% level. Similarly, the F statistic for the double threshold is 12.32, exceeding the crucial value of 10.208 and indicating the statistical importance of the double threshold at the 5% confidence level. However, the F statistics for the triple threshold are not statistically significant, as shown by the p value of 0.393. The above results refute the null hypothesis and indicate a double threshold in the influence of the GEA on green innovation in firms, hence corroborating the hypothesis of a nonlinear association. Table 3 shows that the threshold effect emerges when the GEA reaches 51 and 104.

**Table 2:** Test for multiple threshold models.

Threshold	F Statistics	Probability	Crit10	Crit5	Crit1
Single	11.24	0.007	7.893	8.771	10.703
Double	12.32	0.030	7.524	10.208	15.085
Triple	8.33	0.393	16.289	19.632	24.518

Note: CI: confidence interval; Threshold estimator (CI ¼ 95%) with 300 bootstrap estimates.

**Table 3:** Estimation of thresholds.

Model	Threshold	95% CI	
		Lower	Upper
Single threshold model	51	48.500	52.500
Double threshold model	104	65.000	114.000

Note: CI: confidence interval. Threshold estimator (CI ¼ 95%) with 300 bootstrap estimates.

The aforementioned results confirm the existence of a double threshold effect, and model (2) can be implemented as follows:

$$\begin{array}{l} {GA}_{it}=\alpha_{0}+\alpha_{1}{GEA}_{kt}I({GEA}_{kt}\leq 51)+\alpha_{2}{GEA}_{kt}I({51<GEA}_{kt}\leq 104)\\ \;+{\alpha_{3}{GEA}_{kt}I({GEA}_{kt}>104)+\beta }_{1}Size+\beta_{2}Age+\beta_{3}CR+\beta_{4}ROA\\ \;+\beta_{5}ITR+\beta_{6}Growth+u_{i}+\varepsilon_{it} \end{array}$$ (5)

In Model (5), our sample is separated into three regimes based on the GEA and its 51 and 104 regimes. The comprehensive estimation results are reported in Table 4.

**Table 4:** Full double-threshold regression estimates.

Variable	Description	Coefficient	Standard error
Control variables			
Age	Length of the existence of the enterprises	0.0389***	0.0044
Size	Logarithm of enterprises’ total assets	0.4993***	0.0242
CR	Ratio of current assets to current liabilities	0.0013	0.0040
ROA	Ratio of total revenue to total assets	0.0510	0.1430
ITR	Ratio of operating costs to average inventory	-2.49e-06	3.16e-06
SGR	Product of return on equity and retained earnings ratio	0.0018	0.0016
Explanatory variables			
GEA	Government’s environmental attention below the first threshold (*GEA*≤51)	0.0063***	0.0014
GEA	Government’s environmental attention between the first and second threshold (51<*GEA*≤104)	0.0028***	0.0008
GEA	Government’s environmental attention beyond the second threshold (*GEA*>104)	0.0010	0.0008
Constant		-10.6522***	0.4874
Firm fixed effect		Yes	Yes
Observations		5976	5976

Note: The standard errors are bootstrapped (300 reps). The number of stars is in the order of decreasing statistical significance: ***1%, **5%, and *10%.

As shown in Table 4, the coefficients of the GEA demonstrate that an increase in the GEA exerts a promotional effect on green innovation in firms. The change in the coefficients of the GEA shows that the promotional effect diminishes as the GEA grows. This result verifies the nonlinearity effect of the GEA on corporate green innovation. In addition, the coefficients of the control variables Age and Size are strongly positive at the 1% level, which coincides with reality: The longer a cleantech firm has been established, the more mature its governance structure is, and the more conducive it is to its green R&D activities. In addition, a larger enterprise also means a weaker resource constraint, which benefits cleantech enterprises in carrying out green innovation.

### Unconditional quantile regression results

**Fig 1:**
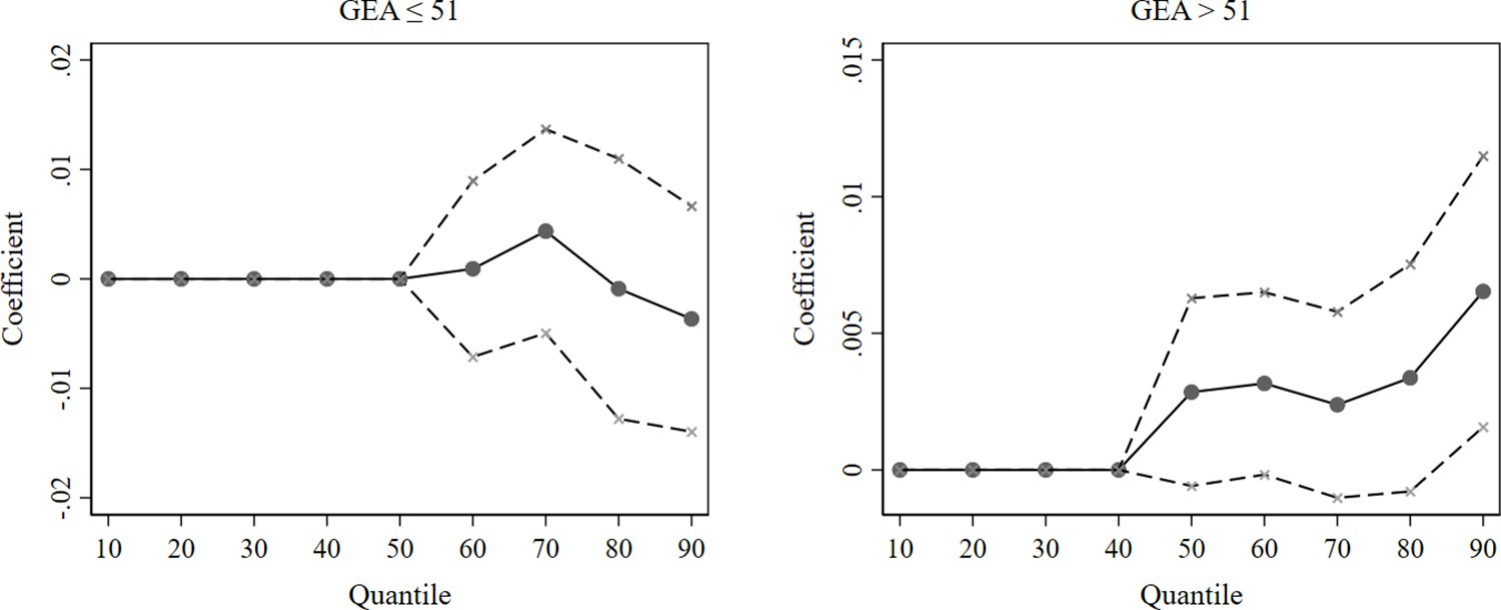
Unconditional quantile regression estimates of the GEA. The solid points are approximations of the influence of the GEA on green innovation in cleantech firms at each quantile; the dashed lines indicate the span of the 95% confidence interval.

We apply 1000 bootstrap replications for our unconditional quantile regression in deriving our estimates and standard errors. Since the regression coefficient of the GEA is insignificant when the GEA is above 104, we divide the sample into two regimes and follow up with unconditional quantile regressions for the first (*GEA*≤51) and second (*GEA*>51) regimes. We focus on the impact of the GEA and only present the estimated coefficients of the GEA on green innovation in cleantech firms in Fig 1.

Fig 1 demonstrates that the impact of the GEA on corporate green innovation fluctuates depending on the degree of corporate innovation. Regardless of whether it is in the first (*GEA*≤51) or second (*GEA*>51) regime, the coefficients of the GEA in the lower quantile regressions (*τ*≤0.4) are minimal and almost equal to zero. To save space, we only discuss the results for the GEA variable reported in Table 5.

**Table 5:** The results of unconditional quantile regressions.

Quantile	GEA ≤ 51		GEA>51	
	Coefficient	Standard error	Coefficient	Standard error
τ = 1	1.10e-34	-7.73e-35	-7.16e-35**	-3.39e-35
τ = 2	2.21e-34	-2.71e-34	-1.43e-34**	-6.92e-35
τ = 3	-4.87e-35	-2.82e-34	-1.06e-34	-9.72e-35
τ = 4	4.42e-34	-3.98e-34	-2.86e-34**	-1.31e-34
τ = 5	-7.22e-34	-5.89e-34	0.00285	-0.00201
τ = 6	0.000921	-0.00527	0.00317	-0.00209
τ = 7	0.00436	-0.00557	0.00238	-0.00206
τ = 8	-0.000894	-0.00735	0.00337	-0.00248
τ = 9	-0.00366	-0.00612	0.00653**	-0.00325

Note: The standard errors are bootstrapped (1000 reps). Our estimates cover 21 years of annual data for 634 cleantech firms. The number of stars is in the order of decreasing statistical significance: ***1%, **5%, and *10%.

Table 5 shows that on either side of the threshold, the coefficient values for the GEA in the lower quantile regressions are minimal, and many are insignificant, which suggests that the GEA has little impact on cleantech firms with low levels of green innovation. From the previous analysis, we conclude that the GEA promotes green innovation in cleantech firms, and this positive effect diminishes as the GEA grows. The results from the quantile regression further indicate that cleantech firms have almost no benefit from the GEA at lower levels of green innovation. Energizing cleantech firms at lower levels of green innovation is essential for enhancing competition in green product markets, often for small and medium-sized enterprises (SMEs). These firms are vulnerable to severe resource constraints and often at a competitive disadvantage, making government support even more necessary. However, even in the first regime (*GEA*≤51), where the positive effect is more substantial, cleantech firms at the lower levels of green innovation receive almost no benefits from the GEA. According to our above analysis, for firms at lower levels of innovation, their weak ability to transform resources into a profit advantage makes the positive impact less pronounced; in addition, their heavier burden from enhanced environmental regulation makes the negative impact more pronounced. This dual interaction leads to firms with lower levels of green innovation benefiting poorly from increasing the GEA.

## Robustness tests

In this section, our study conducts three tests to verify the robustness of our empirical results. At first, we conduct a Davidson-Mackinnon test. The p-value of the test is 0.28, which does not reject the original hypothesis, indicating that GEA is strongly exogenous. In each robustness test, the test results for the single threshold model consistently reject the null hypothesis, indicating the presence of a single threshold in the influence of the GEA on green innovation in cleantech firms. The complete multiple threshold test results in this section are shown in S1 Appendix, and the estimation results are shown in Table 6.

**Table 6:** Robustness results: Threshold regression estimation results.

Variable	Alternative explanatory variable	Adding city-level control variables	Regressions for the post-2003 sample	Deletion of some enterprise samples by condition
Control variables				
Age	0.0378***	-0.0040	0.0401***	0.0123**
	(0.0045)	(0.0085)	(0.0044)	(0.0057)
Size	0.4969***	0.4940***	0.4931***	0.6049***
	(0.0242)	(0.0249)	(0.0244)	(0.0338)
CR	0.0019	-0.0014	0.0012	0.0064
	(0.0040)	(0.0040)	(0.0040)	(0.0065)
ROA	0.0542	0.0274	0.0529	0.0378
	(0.1430)	(0.1431)	(0.1432)	(0.1617)
ITR	-2.46e-06	-2.65e-06	-2.57e-06	2.29e-5
	(3.16e-06)	(3.15e-06)	(3.16e-06)	(5.1e-5)
SGR	0.0018	0.0020	0.0018	0.0016
	(0.0016)	(0.0016)	(0.0016)	(0.0017)
CGDP		0.0532		
		(0.1127)		
CTL		-0.0172		
		(0.0281)		
CEL		0.3361***		
		(0.0788)		
Explanatory variables				
Regime(1)	0.0045**	0.0052***	0.0049***	0.0071***
	(0.0018)	(0.0013)	(0.0013)	(0.0016)
Regime(2)	0.0030**	0.0018***	0.0018**	0.0029**
	(0.0012)	(0.0007)	(0.0007)	(0.0010)
Regime(3)	-0.0010			
	(0.0013)			
Constant	-10.4997***	-15.3694***	-10.4717***	-12.4333***
	(0.4867)	(1.4760)	(0.4902)	(0.6913)
Firm Fixed Effect	Yes	Yes	Yes	Yes
Observations	5976	5878	5941	4562

Note: The standard errors are bootstrapped (300 reps).

The number of stars is in the order of decreasing statistical significance: ***1%, **5%, and *10%.

For the single-threshold model, Regime (1) refers to the interval in which the GEA is below the threshold, and Regime (2) refers to the interval in which the GEA is above the threshold. For the double threshold model, Regime (1) refers to the interval in which the GEA is below the first threshold, Regime (2) refers to the interval in which the GEA is between the first and second thresholds, and Regime (3) refers to the interval in which the GEA is above the second threshold.

### Alternative explanatory variable

**Fig 2:**
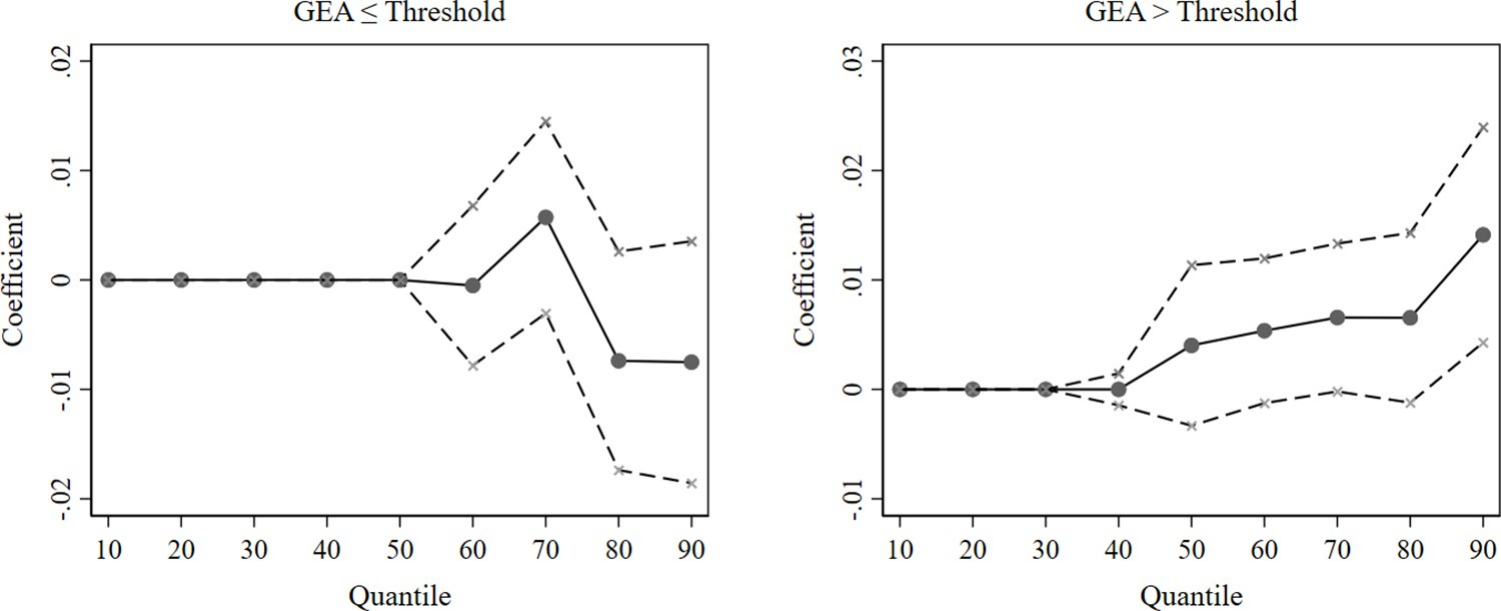
Unconditional quantile regression estimates of the GEA (alternative explanatory variable). The solid points are approximations of the influence of the government’s environmental attention on green innovation in cleantech firms at each quantile; the dashed lines indicate the span of the 95% confidence interval.

In this section, we reconstruct the explanatory variable by narrowing the seed words to 37 words representing “environmental protection.” Table 6 shows the threshold regression results after replacing the explanatory variable, which support our hypothesis of the nonlinear relationship between the GEA and corporate green innovation. Additionally, the estimated coefficients from the unconditional quantile regression and 95% confidence intervals for each quantile are illustrated in Fig 2, confirming that the impact of the GEA on green innovation varies across firms and that cleantech firms have almost no benefit from the GEA at lower levels of green innovation. On this basis, our results are relatively robust.

### Adding new control variables

**Fig 3:**
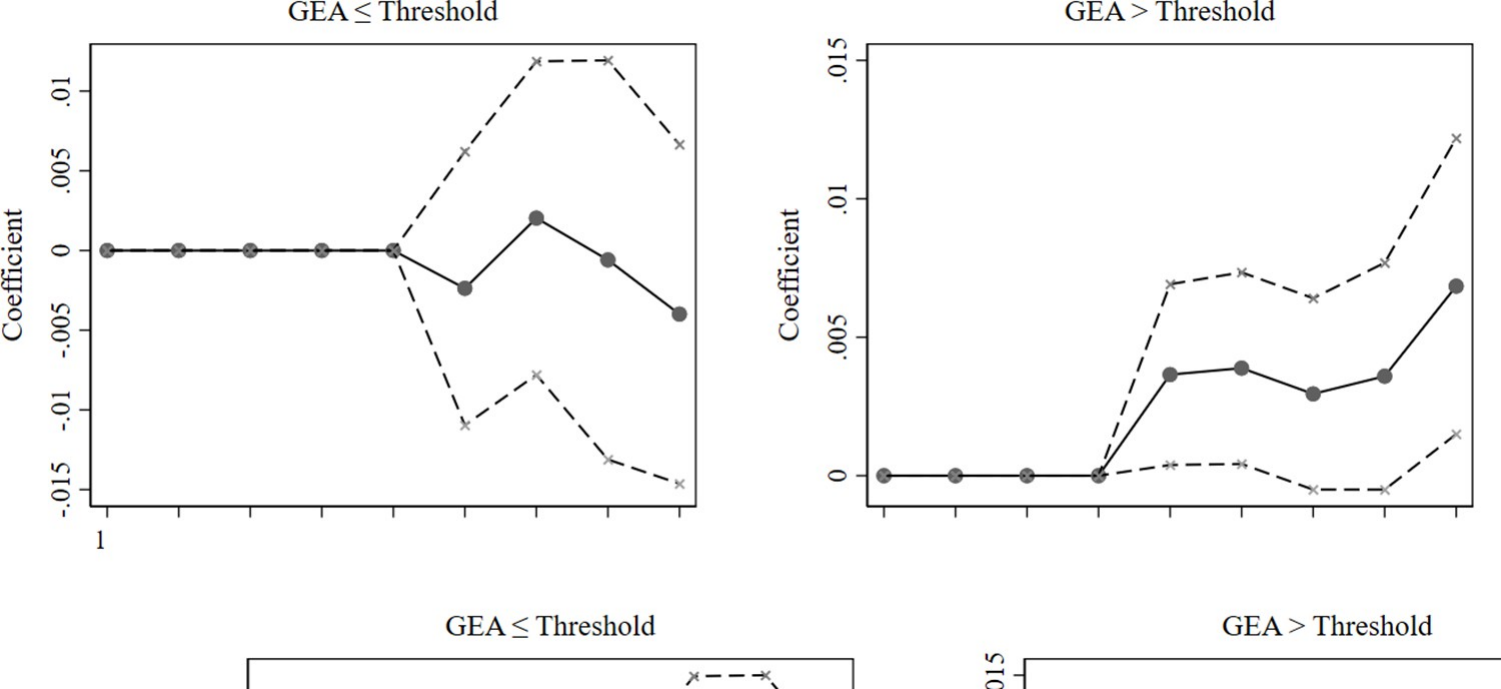
Unconditional quantile regression estimates of the GEA (adding new control variables). The solid points are approximations of the influence of the government’s environmental attention on green innovation in cleantech firms at each quantile; the dashed lines indicate the span of the 95% confidence interval.

In this section, we add several city-level control variables in reference to the literature [48]. We choose to include the following macro variables in the robustness regressions: (1) city economic level (CGDP) denoted by the logarithm of the city-level gross domestic product; (2) city technology level (CTL) denoted by the logarithm of the expenditures in the science of cities; and (3) city education level (CEL) denoted by the logarithm of the education expenditures of cities. We obtain new city-level data from the National Bureau of Statistics of China (NBSC). The results of the threshold regression with the addition of city-level control variables are displayed in Table 6. The conclusion that the positive effect of the GEA diminishes as it grows remains robust. Similarly, the estimated coefficients from the unconditional quantile regression and 95% confidence intervals for each quantile are illustrated in Fig 3, which also indicate that the promotional effect of an increase in the GEA on corporate green innovation is weaker among firms at lower levels of innovation. On this basis, our results are relatively robust.

### Regressions for the post-2003 sample

**Fig 4:**
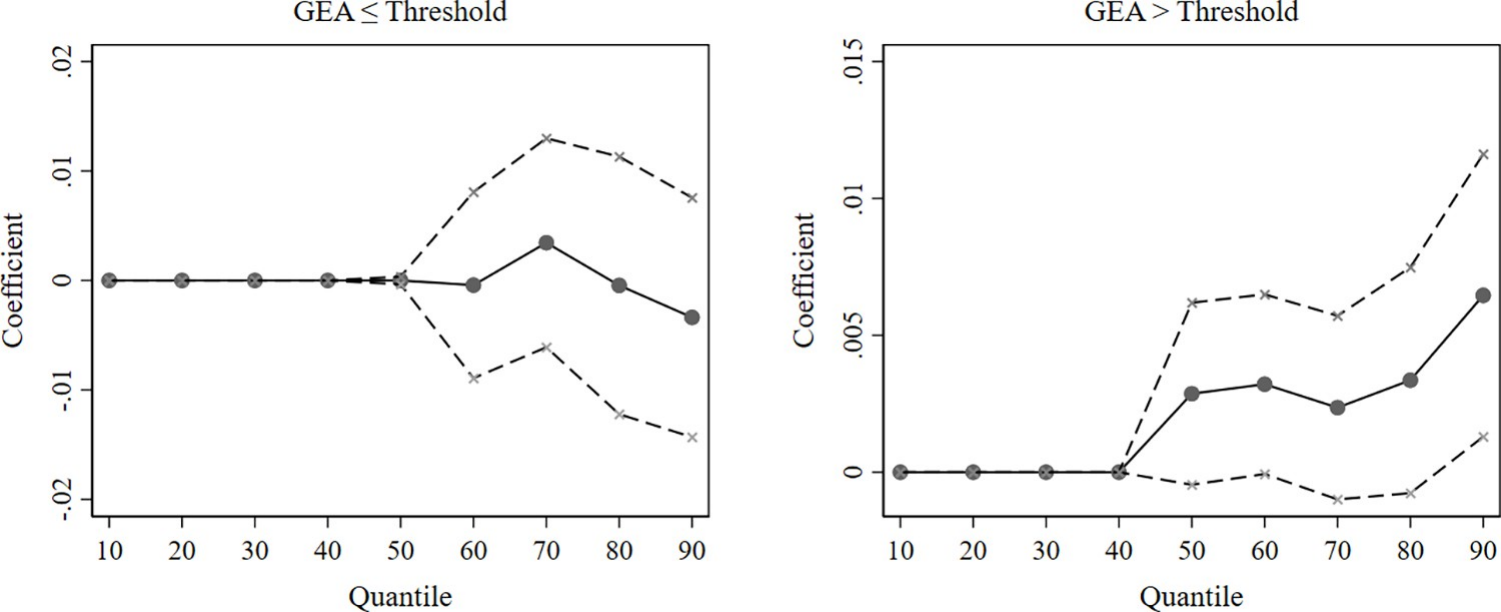
Unconditional quantile regression estimates of the GEA (adding new control variables). The solid points are approximations of the influence of the government’s environmental attention on green innovation in cleantech firms at each quantile; the dashed lines indicate the span of the 95% confidence interval.

In China, the government has not always paid the same attention to the environment as it has to economic growth and development. China has experienced a long period of development in which GDP growth was the primary goal. Not until 2003, when President Hu Jintao put forward *the Scientific Outlook on Development* was “the harmonious development of human beings and nature” considered an essential requirement for the state to promote the reform and development of various undertakings. In this section, we empirically examine the post-2003 data as a further robustness test. From the threshold regression results shown in Table 6, our conclusion that the promotion effect of the GEA diminishes as it grows remains robust. The estimated coefficients from the unconditional quantile regression and 95% confidence intervals for each quantile are illustrated in Fig 4, which reveal that cleantech firms have almost no benefit from the GEA at lower levels of green innovation. On this basis, our results are relatively robust.

### Deletion of some enterprise samples by condition

**Fig 5:**
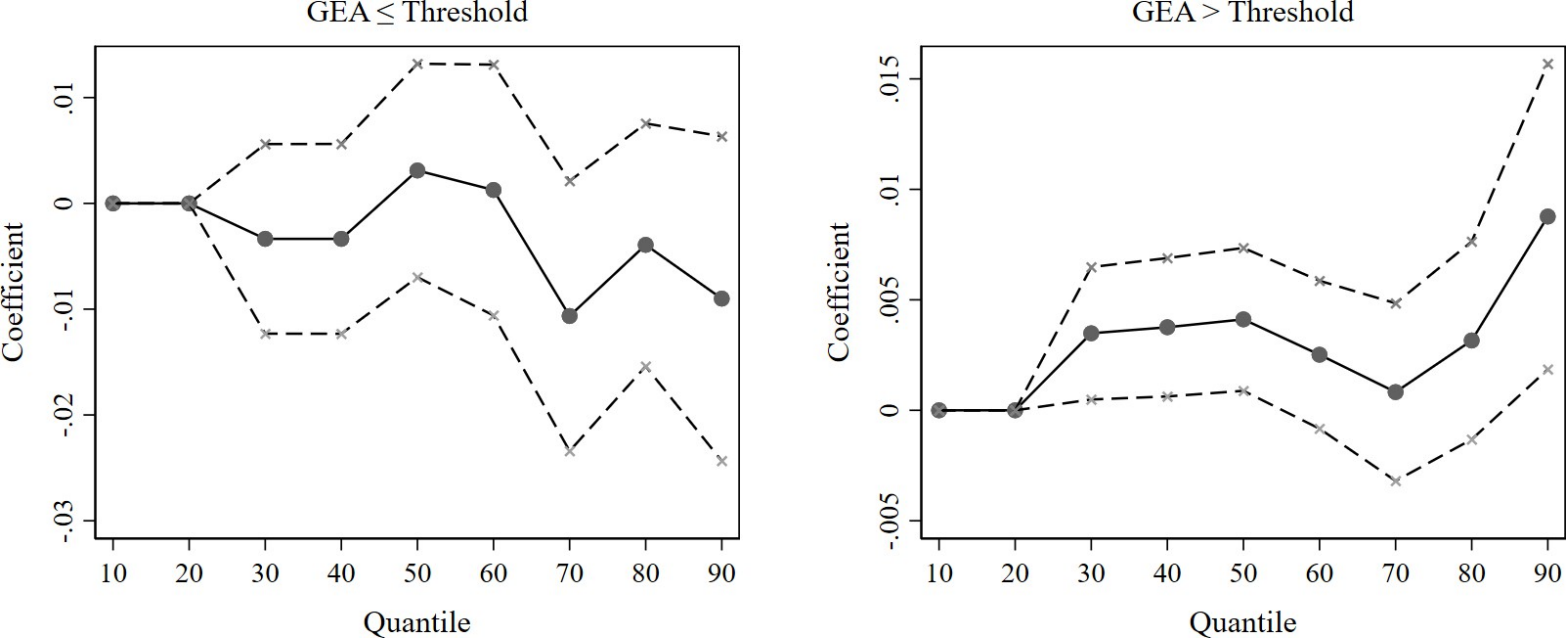
Unconditional quantile regression estimates of the GEA (Deletion of some enterprise samples by condition). The solid points are approximations of the influence of the government’s environmental attention on green innovation in cleantech firms at each quantile; the dashed lines indicate the span of the 95% confidence interval.

In this section, we add a robustness test that considers the effect of patent data structure. Patent data can easily form clusters at 0. To alleviate the impact of the resulting data structure problem on the validity of the empirical results, we delete the sample enterprises whose patent data are all at 0 during the sample time and, conduct the threshold and quantile regression analysis again, respectively, and obtain results consistent with the benchmark regression. The results are illustrated in Table 6 and Fig 5.

## Further analysis

Governments tend to supply various support policies due to the externalities of the research activities [49], and enterprises tend to innovate to pander to the government rather than improve their technological competitiveness [50]. Based on different objectives, enterprises’ innovation determinations can be divided into substantive innovation, aimed at promoting technological progress and maintaining competitive advantage, and strategic innovation, motivated by the purpose of obtaining other benefits [51]. Similarly, corporate green innovation can be divided into substantive green innovation (GAst) and strategic green innovation (GAsg) [52]. The former eventually delivers high-quality green invention patents, and the latter delivers green utility model patents that improve existing clean technologies or products.

In further analysis, we examine the differences in the impact of the GEA on two types of green innovation in cleantech firms. Table 7 illustrates the results of the threshold regressions given two types of green innovation for the explanatory variable. Similar to the threshold regression results for overall green innovation, the GEA has a facilitating impact on strategic green innovation, which diminishes as the GEA grows. In contrast to the previous results, the estimated coefficients of the GEA in the threshold regression on substantive green innovation are negative in both regimes, which shows that the GEA significantly inhibits substantive green innovation in cleantech firms. As the GEA increases, the fact that cleantech firms are prone to placing more emphasis on noninvention patents does not change. In other words, the GEA has contributed to only increased quantity rather than the quality of green innovation for cleantech companies. However, the change in the absolute value of the GEA coefficient shows that both the facilitating and inhibiting effects diminish as the GEA increases.

**Table 7:** Full single-threshold panel regression estimates.

Variable	GAst		GAsg	
	Coefficient	Standard error	Coefficient	Standard error
Control variables				
Age	0.0330***	0.0041	0.0295***	0.0042
Size	0.4269***	0.0225	0.3580***	0.0233
CR	-0.0013	0.0760	0.0009	0.0037
ROA	0.1110	0.1423	0.0556	0.1444
ITR	-2.03e-06	2.91e-06	-1.00e-06	2.91e-06
SGR	0.0003	0.0014	0.0018	0.0015
Threshold variable				
GEA ≤ Threshold	-0.0046***	0.0010	0.0062***	0.0012
GEA > Threshold	-0.0024***	0.0007	0.0026***	0.0007
Constant	-8.8505***	0.4516	-7.7035***	0.4701
Firm fixed effect	Yes		Yes	
Observation	5452		5570	

Note: The standard errors are bootstrapped (300 reps). The number of stars is in the order of decreasing statistical significance: ***1%, **5%, and *10%.

We also test for heterogeneity in the impact of the GEA on cleantech firms with different levels of substantive and strategic green innovation. The comprehensive results of the unconditional quantile regressions are shown in S1 Appendix, and the estimated coefficients are visualized in Fig 5.

**Fig 6:**
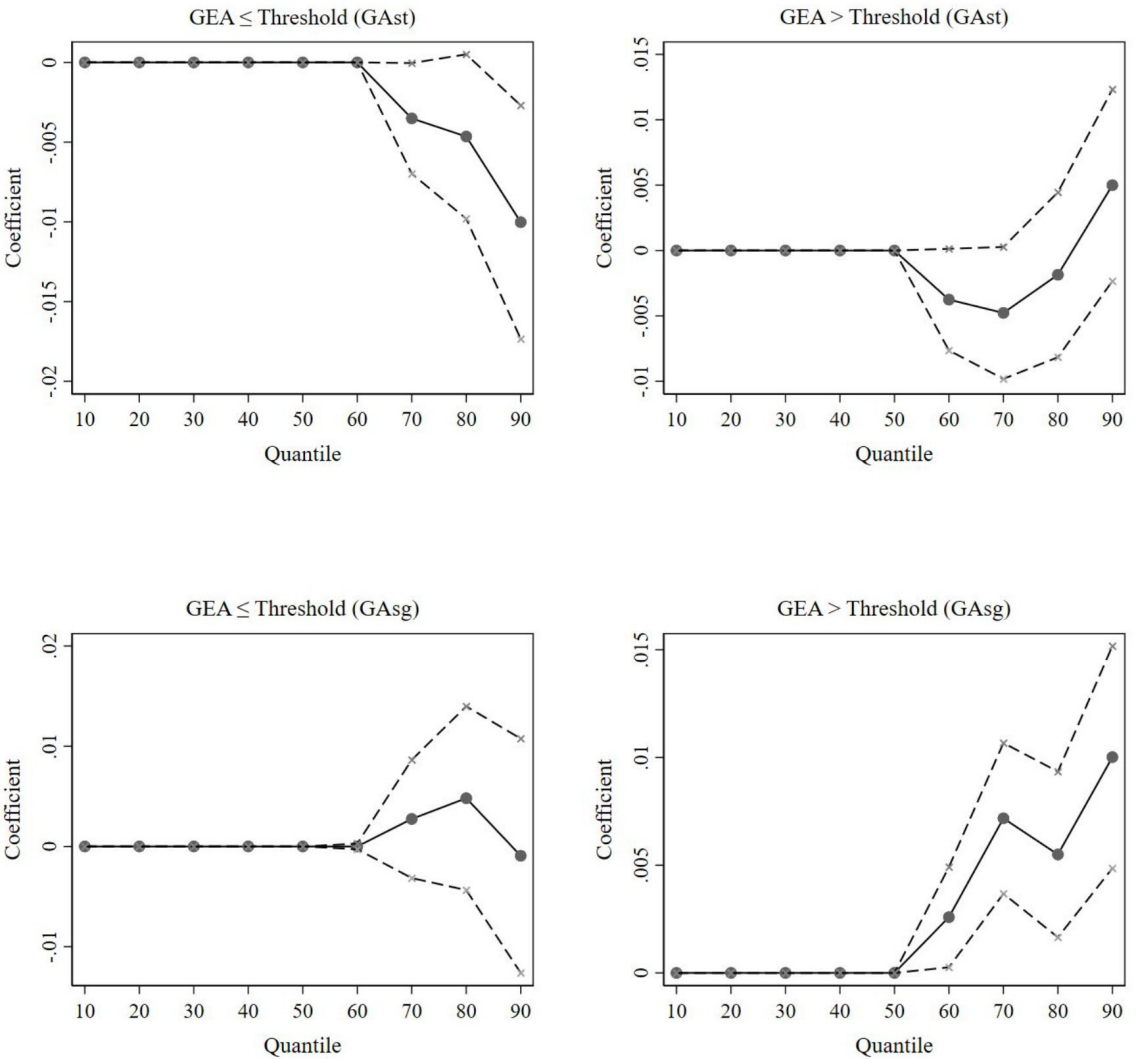
Unconditional quantile regression estimates of the GEA (further analysis). The solid points are approximations of the influence of the government’s environmental attention on green innovation in cleantech firms at each quantile; the dashed lines indicate the span of the 95% confidence interval.

Fig 6 shows that the impact of the GEA on cleantech firms with lower levels of green innovation (*τ*≤0.5) is not significant for either type of green innovation behavior. That is, from the perspective of innovation structure, the GEA does not have an impact on substantive or strategic green innovation in cleantech firms with lower levels of green innovation, which is consistent with our previous conclusion that these firms poorly benefit from the GEA. For firms with higher levels of green innovation, high GEA notably facilitates strategic green innovation, which contradicts the expectation that these firms should lead the way in technological innovation.

## Conclusion

The principal aim of this study is to provide a nonlinear expansion of the previously identified correlation. Furthermore, we assess whether this nonlinearity holds true across the entire spectrum of green innovation of corporations. To achieve this objective, we utilize threshold regression and unconditional quantile regression techniques and obtain compelling findings utilizing an imbalanced dataset consisting of 634 cleantech firms listed in 205 cities from 2001 to 2021. The findings from the threshold regression analysis indicate the presence of a nonlinear relationship between the GEA and green innovation in cleantech firms; specifically, in our sample, an increase in the GEA tends to promote green innovation in firms less when it is above the threshold values 51 and 104. Second, we find that the GEA has a heterogeneous impact on firms with different levels of green innovation. The quantile regression results show that the GEA poorly benefits the green innovation of cleantech firms with low levels of green innovation, even in intervals in which the promotional impact is more substantial. Third, the GEA significantly facilitates strategic green innovation in cleantech firms but inhibits their substantive innovation, and these effects diminish as GEA increases. The quantile regression results show that neither substantive green innovation nor strategic one in cleantech firms suffer from significant effects of GEA at lower levels of green innovation. However, the GEA significantly facilitates strategic green innovation in firms at higher levels of green innovation.

These discoveries have several policy implications. First, we observe that the marginal benefit of the GEA on green innovation wanes beyond certain levels, illustrating the importance of moderating government intervention. The government should rationally allocate its attention and moderately intervene in the environmental sector to effectively promote green innovation in enterprises, enhancing the efficiency of environmental improvement. The government should take measures to promote the application and integration of digital technology in the cleantech industry [53, 54]. Second, policy-makers should give some policy tilt to cleantech firms at lower levels of green innovation, such as reducing environmental regulatory requirements and facilitating more financial support, as energizing their interest in green innovation is essential for enhancing market competition and promoting overall green technological advancement. Third, the government should encourage cooperation in green innovation between cleantech enterprises at higher levels of green innovation and those at lower levels of green innovation to unleash the “coupling effect” of collaborative innovation. Fourth, to improve the quality of green innovation, policy-makers should take targeted measures to stimulate substantive corporate green innovation, such as green credit and green innovation subsidies [20].

Due to limited data availability, we focus solely on listed cleantech firms in this research. Consequently, future research should incorporate the analysis of non-listed cleantech firms and organizations operating in areas outside of clean technology, if feasible. Besides, we only use firms’ green invention patent application data to measure their substantive innovation output level and analyze the impact of GEA on high-quality corporate green innovation. Future research could consider using other metrics, such as green patent citation data, to analyze the impact of GEA on the quality of firms’ green innovation.

## Supporting information

S1 Appendix (DOCX)

S1 Dataset (XLSX)
