# Supplementary material for: The government’s environmental attention and corporate green innovation: A threshold analysis and quantile regression approach
Source: PLoS One. 2024 Oct 31;19(10):e0311154. doi: 10.1371/journal.pone.0311154 (PMC11527151; doi:10.1371/journal.pone.0311154)
Supplement: S1 Appendix — (DOCX) [file pone.0311154.s001.docx]

# Appendix A: Complete construction process of GEA

We construct the variable GEA by following these steps: First, we identify 13 words related to the environment in the General Provisions of the Law of the People’s Republic of China on Environmental Protection as seed words, including “environmental protection,” “pollution control,” “ecological civilization” and so on. Then, we constantly search for similar words by importing the seed words into the Chinese government text database, a featured database of the WinGo Textual Analytics Database. We keep correcting the seed words based on their similarity in this process. Finally, we identified 111 keywords reflecting the government’s environmental attention. After identifying the keywords, we re-import them into the database to obtain the number of occurrences of the keywords in each city in each year, i.e., the value of governmental environmental attention, denoted by GEA. The complete keywords are shown in S1 Table 1.

**S1 Table 1. Keywords to construct the variable GEA.**

| Keywords in English | Keywords in Chinese |
| --- | --- |
| white pollution | 白色污染 |
| atmospheric pollution | 大气污染 |
| atmospheric pollutants | 大气污染物 |
| source of atmospheric pollution | 大气污染源 |
| nitrogen oxide | 氮氧化合物 |
|  | 氮氧化物 |
| low carbon | 低碳 |
| point source | 点源 |
| sulfur dioxide (SO_2_) | 二氧化硫 |
| precautionary | 防范 |
| preventive controls | 防范控制 |
| exhaust | 废气 |
| drain water | 废水 |
| waste liquids | 废液 |
| industrial waste product | 废渣 |
| incinerate | 焚烧 |
| dust | 粉尘 |
| coal ash | 粉煤灰 |
| powder | 粉体 |
| volatile organic compound (VOC) | 挥发性有机物 |
| abatement | 减排 |
| straw | 秸杆 |
| coal dust | 煤尘 |
| coals | 煤炭 |
| surface pollution | 面源污染 |
| discharge | 排放 |
| ventilate | 排气 |
| emission | 排污 |
| the three wastes (e.g. sewage) | 三废 |
| The enterprise that meets the following conditions: (1) does not meet the industrial policy; (2) does not meet the local industrial layout planning, not for industrial information, development and reform, land, planning, environmental protection, industry and commerce, quality supervision, safety supervision, electricity, and other related approval procedures; (3) unable to stable to meet the standard emissions of enterprises. | 散乱污 |
| water contamination | 水污染 |
| plastic products | 塑料制品 |
| desulphurization | 脱硫 |
| denitrification | 脱硝 |
| waste remaining after processing ore | 尾矿 |
| waste gas from the engine (e.g. exhaust) | 尾气 |
| greenhouse gas | 温室气体 |
| contaminate | 污染 |
| contamination | 污染物 |
| pollutant source | 污染源 |
| pollution control | 污染治理 |
| sewage disposal | 污水排放 |
| haze | 雾霾 |
| smoke and dust | 烟尘 |
| flue gas | 烟气 |
| gale | 扬尘 |
| odor | 异味 |
| lampblack | 油烟 |
| hazardous gas | 有害气体 |
| dirty and disordered | 脏乱 |
| noises | 噪声 |
|  | 噪音 |
| anti-pollution | 治污 |
| priority sewage disposal | 重点排污 |
| heavily polluting industries | 重污染行业 |
| energy storage | 储能 |
| consume energy | 耗能 |
| reduce consumption | 降耗 |
| energy conservation | 节能 |
| sustainable development | 可持续发展 |
| energy audits | 能源审计 |
| graphene | 石墨烯 |
| water resources | 水资源 |
| solar power | 太阳能 |
| new materials | 新材料 |
|  | 新型材料 |
| new energy | 新能源 |
| new construction materials | 新型建材 |
| circulate | 循环 |
| recycle | 循环利用 |
| renewable resource | 再生资源 |
| resource | 资源 |
| resource protection | 资源保护 |
| resource conservation | 资源节约 |
| odor | 臭气 |
| ozone (O_3_) | 臭氧 |
| magnanimity | 大气 |
| atmospheric environment | 大气环境 |
| ground settlement | 地面沉降 |
| improve the soil | 改土 |
| system of river chiefs | 河长制 |
| eco-friendly | 环保 |
| environment | 环境 |
| environmental protection | 环境保护 |
| air | 空气 |
| blue sky | 蓝天 |
| war on blue sky | 蓝天保卫战 |
| green | 绿色 |
| green economy | 绿色经济 |
| green hills and clear waters | 绿水青山 |
| lightweight | 轻量化 |
| cleanse | 清洁 |
| habitat environment | 人居环境 |
| ecology | 生态 |
| ecological protection | 生态保护 |
| ecological environment | 生态环境 |
| ecological construction | 生态建设 |
| ecological civilization | 生态文明 |
| ecological civilization construction | 生态文明建设 |
| ecosystems | 生态系统 |
| water environment | 水环境 |
| headwaters of a river | 水源地 |
| water quality | 水质 |
| restoring agricultural land forest | 退耕还林 |
| liveable | 宜居 |
| source of drinking water | 饮用水源 |
|  | 饮用水源地 |
| river management | 治河 |
| mountain management | 治山 |
| water management | 治水 |
| nature | 自然 |

# Appendix B: Tests for multiple thresholds model in Robustness tests

The test results for multiple threshold models after reconstructing the explanatory variable are shown in S1 Table 2. As presented in Appendix Table 2, the F statistics for the single threshold is 15.36, which is higher than the critical value of 9.059, suggesting the statistical significance of the single threshold at 5%. Similarly, the F statistics for the double threshold is 9.27, which is higher than the critical value of 8.023, suggesting the statistical significance of the double threshold at 10%. The above results reject the null hypothesis and suggest the existence of double threshold in the impact of GEA on corporate green innovation, thereby supporting the hypothesis of a nonlinear relationship. S1 Table 3 shows the threshold effect occurs when the government’s environmental attention reaches 37 and 67.

**S1 Table 2. Test for multiple thresholds models (Alternative explanatory variable).**

| Threshold | *F* Statistics | Probability | Crit10 | Crit5 | Crit1 |
| --- | --- | --- | --- | --- | --- |
| Single | 15.36 | 0.003 | 7.490 | 9.059 | 11.985 |
| Double | 9.27 | 0.077 | 8.023 | 11.036 | 17.303 |
| Triple | 12.28 | 0.447 | 22.890 | 27.096 | 37.513 |

CI: confidence interval; Threshold estimator (CI ¼ 95%), with 300 bootstrap estimates.

**S1 Table 3.** Estimation of thresholds (Alternative explanatory variable).

| Model | Threshold | Lower (95%CI) | Upper (95% CI) |
| --- | --- | --- | --- |
| Th-1 | 67.0000 | 63.0000 | 73.0000 |
| Th-2 | 37.0000 | 33.5000 | 38.0000 |

CI: confidence interval. Threshold estimator (CI ¼ 95%), with 300 bootstrap estimates.

The test results for multiple threshold models after adding new control variables are shown in S1 Table 4. As presented in S1 Table 4, the F statistics for the single threshold is 21.33, which is higher than the critical value of 11.901, suggesting the statistical significance of the single threshold at 1%. However, the F statistics for the triple threshold is not statistically insignificant, with p-values of 0.653. The above results reject the null hypothesis and suggest the existence of single threshold in the impact of GEA on corporate green innovation, thereby supporting the hypothesis of a nonlinear relationship. S1 Table 5 shows the threshold effect occurs when the government’s environmental attention reaches 51.

**S1 Table 4. Test for multiple thresholds models (Adding new control variables).**

| Threshold | *F* Statistics | Probability | Crit10 | Crit5 | Crit1 |
| --- | --- | --- | --- | --- | --- |
| Single | 21.33 | 0.000 | 7.484 | 8.936 | 11.901 |
| Double | 3.42 | 0.653 | 8.081 | 9.813 | 12.646 |
| Triple | 8.01 | 0.170 | 10.281 | 12.389 | 16.744 |

CI: confidence interval; Threshold estimator (CI ¼ 95%), with 300 bootstrap estimates.

**S1 Table 5. Estimation of thresholds (Adding new control variables).**

| Model | Threshold | Lower (95%CI) | Upper (95% CI) |
| --- | --- | --- | --- |
| Th-1 | 51.0000 | 48.5000 | 52.0000 |

CI: confidence interval. Threshold estimator (CI ¼ 95%), with 300 bootstrap estimates.

The test results for multiple threshold models for regressions for the post-2003 sample are shown in S1 Table 6. As presented in S1 Table 6, the F statistics for the single threshold is 13.25, which is higher than the critical value of 9.612, suggesting the statistical significance of the single threshold at 5%. However, the F statistics for the triple threshold is not statistically insignificant, with p-values of 0.103. The above results reject the null hypothesis and suggest the existence of single threshold in the impact of GEA on corporate green innovation, thereby supporting the hypothesis of a nonlinear relationship. S1 Table 7 shows that the threshold effect occurs when the government’s environmental attention reaches 51.

**S1 Table 6. Test for multiple thresholds models (Regressions for the post-2003 sample).**

| Threshold | *F* Statistics | Probability | Crit10 | Crit5 | Crit1 |
| --- | --- | --- | --- | --- | --- |
| Single | 13.25 | 0.007 | 8.099 | 9.612 | 12.584 |
| Double | 7.51 | 0.103 | 7.514 | 9.182 | 12.364 |
| Triple | 5.15 | 0.557 | 12.808 | 16.608 | 23.022 |

CI: confidence interval; Threshold estimator (CI ¼ 95%), with 300 bootstrap estimates.

**S1 Table 7. Estimation of thresholds (Regressions for the post-2003 sample).**

| Model | Threshold | Lower (95%CI) | Upper (95% CI) |
| --- | --- | --- | --- |
| Th-1 | 51.0000 | 48.5000 | 58.5000 |

CI: confidence interval. Threshold estimator (CI ¼ 95%), with 300 bootstrap estimates.

# Appendix C: Tests for multiple thresholds model in further analysis

The test results for multiple threshold models after changing the explained variable to GAst (substantive green innovation) are shown in S1 Table 8. The results in S1 Table 8, reject the null hypothesis and suggest the existence of double threshold in the impact of GEA on corporate substantive green innovation, thereby supporting the hypothesis of a nonlinear relationship. However, the coefficients of GEA in the double threshold model are insignificant, which means the impact of the threshold points are not significant enough in the divided regimes or the quality of the data is insufficient to detect the presence of the thresholds significantly. The coefficients of GEA in the single threshold model are significant in both regimes, supporting the existence of single threshold in the impact of GEA on corporate substantive green innovation. S1 Table 9 shows the threshold effect occurs when the government’s environmental attention reaches 65. Similar to the above results, a single threshold model exists in the impact of GEA on corporate strategic green innovation (GAsg), and the threshold value of GEA is 51.

**S1 Table 8. Two types of green innovation: tests for multiple thresholds model.**

| Explained variable | Threshold Model | F Statistics | Probability | Crit10 | Crit5 | Crit1 |
| --- | --- | --- | --- | --- | --- | --- |
| GAst | Single | 12.31 | 0.013 | 7.946 | 8.866 | 12.480 |
|  | Double | 9.79 | 0.037 | 7.161 | 8.490 | 11.474 |
|  | Triple | 6.87 | 0.260 | 9.816 | 12.170 | 17.908 |
| GAsg | Single | 24.32 | 0.000 | 8.417 | 10.394 | 14.072 |
|  | Double | 11.53 | 0.010 | 7.456 | 8.798 | 11.340 |
|  | Triple | 8.43 | 0.600 | 20.727 | 23.634 | 27.737 |

CI: confidence interval; Threshold estimator (CI ¼ 95%), with 300 bootstrap estimates.

**S1 Table 9.** Two types of green innovation: estimation of thresholds.

| Explained variable | Threshold | 95% CI | |
| --- | --- | --- | --- |
|  |  | Lower | Upper |
| GAst | 65 | 63.000 | 66.000 |
| GAsg | 51 | 48.000 | 64.000 |

CI: confidence interval. Threshold estimator (CI ¼ 95%), with 300 bootstrap estimates.

# Appendix D: Complete unconditional quantile regressions in further analysis

**S1 Table 10.** The results of unconditional quantile regressions (GAst).

| Quantile | *GEA* ≤ 65 | | *GEA*＞65 | |
| --- | --- | --- | --- | --- |
|  | Coefficient | Standard error | Coefficient | Standard error |
| τ=1 | -8.44E-36 | -2.90E-35 | 0 | -3.07E-35 |
| τ=2 | -1.69E-35 | -4.85E-35 | 0 | -6.12E-35 |
| τ=3 | -9.22E-35 | -1.00E-34 | 0 | -8.24E-35 |
| τ=4 | -3.38E-35 | -8.83E-35 | 0 | -1.19E-34 |
| τ=5 | -4.29E-35 | -1.52E-34 | 0 | -1.53E-34 |
| τ=6 | -1.84E-34 | -1.47E-34 | -0.00375* | -0.00226 |
| τ=7 | -0.00351* | -0.00212 | -0.00477 | -0.00304 |
| τ=8 | -0.00465 | -0.00319 | -0.00185 | -0.00393 |
| τ=9 | -0.0100** | -0.00442 | 0.005 | -0.00457 |

The standard errors are bootstrapped (1000 reps). Our estimates cover 21 years of annual data for 634 cleantech firms. The number of stars is in the order of decreasing statistical significance: ***1%, **5%, and *10%.

**S1 Table 11.** The results of unconditional quantile regressions (GAsg).

| Quantile | *GEA* ≤ 51 | | *GEA*＞51 | |
| --- | --- | --- | --- | --- |
|  | Coefficient | Standard error | Coefficient | Standard error |
| τ=1 | 4.84E-35 | -6.57E-35 | -3.16E-35 | -2.05E-35 |
| τ=2 | 9.69E-35 | -1.73E-34 | -6.33E-35 | -4.73E-35 |
| τ=3 | 0 | -1.42E-34 | -1.43e-34** | -5.78E-35 |
| τ=4 | 1.94E-34 | -3.12E-34 | -1.27E-34 | -7.73E-35 |
| τ=5 | -1.58E-34 | -3.66E-34 | 2.88E-36 | -1.04E-34 |
| τ=6 | 0 | -0.000303 | 0.00259* | -0.0015 |
| τ=7 | 0.00274 | -0.00351 | 0.00718*** | -0.00203 |
| τ=8 | 0.00481 | -0.00568 | 0.00550** | -0.00223 |
| τ=9 | -0.00093 | -0.00684 | 0.0100*** | -0.00312 |

The standard errors are bootstrapped (1000 reps). Our estimates cover 21 years of annual data for 634 cleantech firms. The number of stars is in the order of decreasing statistical significance: ***1%, **5%, and *10%.
